# Provisional COVID-19 Age-Adjusted Death Rates, by Race and Ethnicity — United States, 2020–2021

**DOI:** 10.15585/mmwr.mm7117e2

**Published:** 2022-04-29

**Authors:** Benedict I. Truman, Man-Huei Chang, Ramal Moonesinghe

**Affiliations:** ^1^Office of the Director, National Center for HIV, Viral Hepatitis, STD, and TB Prevention, CDC;^ 2^COVID-19 Emergency Response Team; ^3^Strategic Innovative Solutions, LLC., Clearwater, Florida.

Disparities in COVID-19 death rates by race and ethnicity have been reported in the United States (*1*,*2*). In response to these disparities, preventive, medical care, and social service assistance programs were implemented to lessen disparities in COVID-19 outcomes, including grants to support state, tribal, local, and territorial health department responses (*3*). The potential impact of such efforts on annual changes in racial and ethnic disparities in mortality rates that identify COVID-19 as the underlying cause of death has not been previously reported. This analysis used U.S. provisional mortality data from death certificates collected by CDC’s National Vital Statistics System (NVSS) to estimate changes in COVID-19–related age-adjusted death rates (AADRs) by race and ethnicity during 2020–2021. Compared with non-Hispanic multiracial persons (the group with the lowest death rate), significant decreases in AADR ratios occurred during 2020–2021 among non-Hispanic American Indian or Alaska Native (AI/AN) persons (34.0%), non-Hispanic Asian (Asian) persons (37.6%), non-Hispanic Black or African American (Black) persons (40.2%), Hispanic persons (37.1%), and non-Hispanic White (White) persons (14%); a non-statistically significant 7.2% increase in AADR ratio occurred among non-Hispanic Native Hawaiian or other Pacific Islander (NH/OPI) persons. Despite reductions in AADR disparities from 2020 to 2021, large disparities in AADR by race and ethnicity remained in 2021. Providing effective preventive interventions, including vaccination and clinical care, to all communities in proportion to their need for these interventions is necessary to reduce racial and ethnic disparities in COVID-19 deaths.

CDC WONDER* mortality data from 2020 (final) and 2021 (provisional) reported to NVSS as of February 6, 2022, was used to assess annual changes in COVID-19 deaths among U.S. residents of any age during January 2020–December 2021. Cause of death codes from the *International Classification of Diseases, Tenth Revision* (ICD-10) were used to classify diseases as underlying causes of death^†^ (*4*). COVID-19 deaths were defined as deaths for which COVID-19 was listed on the death certificate as a confirmed or presumed underlying cause of death (ICD-10 code U07.1). AADRs and their SEs were downloaded using CDC WONDER Provisional Multiple Cause of Death data file “2018–last month” for numbers of decedents, mid-year resident populations, and crude death rates. The data included COVID-19 deaths by sex (female and male), age group (≤24, 25–44, 45–64, 65–74, and ≥75 years) and race and ethnicity (AI/AN, Asian, Black, Hispanic, NH/OPI, and White persons, and persons who were listed as non-Hispanic more than one race [multiracial]). Deaths that occurred among residents of U.S. territories and foreign countries were excluded.

At the time of this analysis, 2021 U.S. population estimates were unavailable; therefore, midyear U.S. Census Bureau population estimates (as of July 1, 2020) were used to calculate estimated COVID-19-related death rates (deaths per 100,000 population) for 2020 and 2021.^§^ Crude death rates were calculated by sex, age group, and race and ethnicity, and AADRs were calculated by race and ethnicity. Changes in AADR within each racial and ethnic group from 2020 (*5*) to 2021 with 95% CIs and statistical tests of significance were calculated. Using the non-Hispanic multiracial group (the group with the lowest death rate) as the referent group,^¶^ changes from 2020 to 2021 in AADR ratios with 95% CIs and statistical tests of significance of differences between each racial and ethnic group and the referent group were calculated. The referent group consisted of persons who identified with two or more races (e.g., White and Asian, Black and AI/AN, or any other combination of races). Pearson’s chi-square tests of differences in the distribution of decedents by sex, age group, and race and ethnicity were compared with the estimated midyear U.S. Census Bureau population estimate. Z-tests of statistical significance were used to compare differences in the percent change in each measure of relative disparity. P-values <0.05 were considered statistically significant. Statistical analyses were performed using SAS software (version 9.4; SAS Institute). To detect and correct computational errors, the analyses were replicated by two analysts independently. To assess sensitivity of the results to changing the referent group, separate analyses were conducted with multiracial and non-Hispanic White persons as referent groups. This activity was reviewed by CDC and was conducted consistent with applicable federal law and CDC policy.**

Total COVID-19 deaths (and crude death rates) increased from 350,831 (106.5 per 100,00 population) in 2020 to 411,465 (124.9) in 2021 (Table 1). The numbers or percentages of decedents in 2020 and 2021 with missing values was small based on sex (the number was 0 for both years), age group (the numbers were 4 and 2, respectively), and race and ethnicity (0.4% and 0.2%, respectively). Persons who were male (54.9%–56.7%), those aged >65 years (68.0%–80.6%), Black persons (13.3%–16.1%), and White persons (59.6%–65.2%) were significantly overrepresented (p<0.001) among decedents compared with the standard population in both 2020 and 2021.

**TABLE 1:** U.S. resident population and proportional distribution of COVID-19 decedents by sex, age-group, and race/ethnicity, and COVID-19–associated death rates* — United States, 2020–2021

Characteristic	2020 Population	2020 (final)^†^			2021 (provisional)^†^		
		Deaths		P-value	Deaths		P-value
	No.	No.	Column % or rate^§^		No.	Column % or rate	
**Sex**							
Female	167,227,921	158,319	45.1	<0.001**^¶^**	178,028	43.3	<0.001**^¶^**
Male	162,256,202	192,512	54.9		233,437	56.7	
**Age group, yrs**							
≤24	102,849,110	604	0.2	<0.001**^¶^**	1,595	0.4	<0.001**^¶^**
25–44	88,205,838	8,333	2.4		21,550	5.2	
45–64	82,769,810	59,054	16.8		108,838	26.5	
65–74	32,549,398	76,277	21.7		101,408	24.7	
≥75	23,109,967	206,559	58.9		178,072	43.3	
Not stated	NA	4	0.0		2	0.0	
Race/Ethnicity**							
White	196,773,390	209,138	59.6	<0.001**^¶^**	268,121	65.2	<0.001**^¶^**
Hispanic	61,312,879	65,237	18.6		67,922	16.5	
Black	41,427,341	56,383	16.1		54,790	13.3	
Asian	19,367,197	12,693	3.6		12,576	3.1	
AI/AN	2,432,338	4,265	1.2		4,518	1.1	
Multiracial**^††^**	7,557,471	1,055	0.3		1,793	0.4	
NH/OPI	613,507	633	0.2		1,069	0.3	
Not stated	NA	1,427	0.4		676	0.2	
**Total**	**329,484,123**	**350,831**	106.5*	**NA**	**411,465**	124.9*	**NA**

**Abbreviations**: AI/AN = American Indian or Alaska Native; NA = not applicable; NH/OPI = Native Hawaiian or other Pacific Islander.

* Deaths per 100,000 population.

^†^ Proportional distribution and rate for 2020 are final and for 2021 are provisional. At the time of this analysis, 2021 U.S. population estimates were unavailable; therefore, midyear U.S. Census Bureau population estimates as of July 1, 2020 were used to calculate estimated COVID-19–associated death rates for 2020 and 2021.

^§^ This column contains column percent plus the overall total rate in the last row.

^¶^ P-values <0.05 were considered statistically significant (Pearson’s chi-square).

** Hispanic persons could be of any race; AI/AN, Asian, Black, NH/OPI, White, and multiracial persons were non-Hispanic.

^††^ More than one race reported.

In 2020, AADR was lowest among multiracial persons (29.6 per 100,000 population) (Table 2). In 2020, compared with multiracial persons, the AADR ratio (relative disparity) was 5.9 for AI/AN, 2.1 for Asian, 4.8 for Black, 5.3 for Hispanic, 3.8 for NH/OPI, and 2.3 for White persons. Overall AADR increased by 19.2% from 85.0 in 2020 to 101.3 per 100,000 U.S. residents in 2021, including 3.8% among AI/AN, 57.1% among multiracial, 68.3% among NH/OPI, and 35.1% among White persons; and decreased by 1.9% among Asian, 6.1% among Black, and 1.2% among Hispanic persons. In 2021, the AADR relative disparity decreased by 34.0% for AI/AN, 37.6% for Asian, 40.2% for Black, 37.1% for Hispanic, and 14.0% for White persons. The increase among NH/OPI persons was not statistically significant (7.2%, from 3.8 in 2020 to 4.1 in 2021) (Figure). Using non-Hispanic White persons as the referent group yielded significant decreases in AADR ratios for AI/AN (−23.2%), Asian (−27.4%), Black (−30.5%), and Hispanic persons (−26.9%), but a significant increase for NH/OPI persons (24.6%).

**TABLE 2:** Changes in age-adjusted death rates* with COVID-19 as underlying cause, by race/ethnicity^†^ — United States, 2020–2021

Race/Ethnicity	AADR		% Change in AADR, 2020–2021 (95% CI)	RR (95% CI)		% Change in AADR ratio, 2020–2021(95% CI)^§^
	2020	2021		2020	2021	
AI/AN	175.9	182.5	3.8 (−0.7 to 8.2)	5.9 (5.5 to 6.3)	3.9 (3.7 to 4.1)	−34.0 (−39.7 to −28.2)
Hispanic	155.5	153.7	−1.2 (−2.2 to −0.1)	5.3 (4.9 to 5.6)	3.3 (3.2 to 3.5)	−37.1 (−41.9 to −32.3)
Black	142.0	133.4	−6.1 (−7.2 to −4.9)	4.8 (4.5 to 5.1)	2.9 (2.7 to 3.0)	−40.2 (−44.8 to −35.6)
NH/OPI	112.4	189.2	68.3 (51.2 to 85.4)	3.8 (3.4 to 4.2)	4.1 (3.8 to 4.4)	7.2 (−6.4 to 20.7)
White	66.6	90.0	35.1 (34.4 to 35.8)	2.3 (2.1 to 2.4)	1.9 (1.8 to 2.0)	−14.0 (−20.5 to −7.5)
Asian	63.1	61.9	−1.9 (−4.5 to 0.7)	2.1 (2.0 to 2.3)	1.3 (1.3 to 1.4)	−37.6 (−42.6 to −32.6)
Multiracial^§^	29.6	46.5	57.1 (45.2 to 69.0)	Ref.	Ref.	Ref.
**Total**	**85.0**	**101.3**	**19.2 (18.6 to 19.7)**	**NA**	**NA**	**NA**

**Abbreviations:** AADR = age-adjusted death rate; AI/AN = American Indian or Alaska Native; NA = not applicable; NH/OPI = Native Hawaiian or other Pacific Islander; Ref. = referent group; RR = rate ratio.

* Deaths per 100,000 standard population.

^†^ Hispanic persons could be of any race; AI/AN, Asian, Black, NH/OPI, White, and multiracial persons were non-Hispanic.

^§^ More than one race listed.

**FIGURE:**
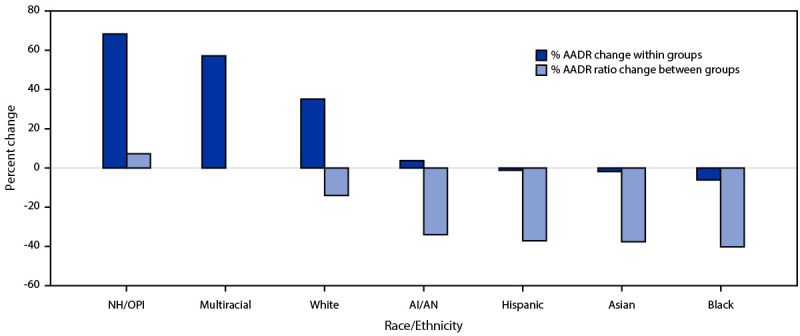
Percent change in COVID-19 age-adjusted death rate and ratio,* by race/ethnicity^†^ — United States, 2020–2021 **Abbreviations:** AADR = age-adjusted death rate; AI/AN = American Indian or Alaska Native; NH/OPI = Native Hawaiian or other Pacific Islander. * Referent group was multiracial persons. ^†^ Hispanic persons could be of any race; AI/AN, Asian, Black, NH/OPI, White, and multiracial persons were non-Hispanic.

## Discussion

Compared with multiracial U.S. residents, who had the lowest COVID-19–related death rate, disparity in COVID-19 AADRs decreased significantly for most racial and ethnic groups from 2020 to 2021. Using multiracial persons as the referent group enabled the presentation of changes in relative disparity for White persons, who accounted for 59.6% of all COVID-19 decedents in 2020 and 65.2% in 2021. Reductions in disparities in AADR for most racial and ethnic groups reflect the widespread impact of effective interventions, including vaccination, deployed since January 2020 to prevent SARS-CoV-2 infection and severe COVID-19 disease and death. Because AADR increased 57.1% among multiracial persons (the referent group for measuring disparity) from 2020 to 2021, percent reductions in AADR ratios for the other racial and ethnic groups were approximately 10% larger than they would have been had non-Hispanic White persons been used as the referent group and larger than they would have been if the AADR for the multiracial group had decreased or remained constant from 2020 to 2021. Low rates of COVID-19 AADR among 33.8 million multiracial persons in 2020 and 2021 are consistent with the lower all-causes mortality experience of components of the multiracial group counted in the 2020 U.S. Census as follows: White persons and another race (57%), White and AI/AN persons (11.8%), White and Asian persons (7.9%), White and Black persons (9.2%), Black persons and another race (3%), and other combinations of race (11%) (*6*). Despite reductions in AADR disparities from 2020 to 2021, large disparities in AADR between each racial/ethnic group remained in 2021 regardless of the referent group chosen.

These findings are consistent with previously published estimates of racial and ethnic disparities in AADR using non-Hispanic White persons as the referent group (*1*,*7*,*8*). This report adds previously unknown changes in relative disparity among White persons, who account for the overwhelming majority of all COVID-19 deaths, emphasizing the critical need for nationwide efforts to reduce AADR among all racial and ethnic groups during 2022 and beyond. These efforts should consider federal, state, tribal, and other local expertise, technical assistance, and resources that deliver preventive interventions, including vaccination and clinical care to all racial and ethnic communities in proportion to their needs rather than their historical demand for or access to services. Through a $2.25 billion national initiative, CDC’s Center for State, Tribal, Local, and Territorial Support (CSTLTS) awarded grants to 108 states, counties (urban and rural), cities, territories, and tribal nations (through separate funding agreements) to implement strategies in collaboration with community-based organizations (*3*), including efforts to improve surveillance; isolation; contact tracing; and the delivery of mobile diagnostic testing, vaccination, and outpatient COVID-19 treatment services in nonclinical settings such as homes, churches and other community-gathering places.

The findings in this report are subject to at least five limitations. First, final 2020 and provisional 2021 data were analyzed in February 2022. Provisional numbers and rates might change as additional information is received and 2021 data are finalized. Second, because the timeliness of death certificate submission from states and local jurisdictions to the NVSS varied by jurisdiction and might also vary by race and ethnicity within a jurisdiction during 2020 and 2021, the national distribution of deaths by race and ethnicity might be affected by measurement bias. Third, certain categories of race (e.g., AI/AN, Asian, and multiracial) and Hispanic ethnicity reported on death certificates might have been misclassified (*9*), possibly resulting in under- or overestimates of death rates for some groups. Fourth, use of the non-Hispanic multiracial category as the referent group, instead of the more precise and statistically stable non-Hispanic White category, might have overestimated the sizes of the relative disparity ratios. Finally, the underlying cause of death for certain persons might have been misclassified because of small differences in availability of diagnostic testing for SARS-CoV-2, the virus that causes COVID-19, by race and ethnicity (*10*). Differential misclassification of the underlying cause of deaths by race and ethnicity might have resulted in an under- or overestimation of COVID-19–associated deaths by race and ethnicity.

During 2020–2021, AADRs decreased for Black (−6.1%), Asian (−1.9%) and Hispanic persons (−1.2%) but increased for NH/OPI (68.3%), multiracial (57.1%), White (35.1%) and AI/AN persons (3.8%). Relative disparities in AADR ratios from COVID-19 decreased significantly for most racial and ethnic groups, including non-Hispanic White persons when compared with multiracial persons who had the lowest AADR in 2020 and 2021. Providing effective preventive interventions, including vaccination and clinical care, to all communities in proportion to their needs can help to decrease racial and ethnic disparities in COVID-19 deaths.

**Summary**

**What is already known about this topic?**

In 2020, racial and ethnic disparities in COVID-19 age-adjusted death rates (AADR) were reported among U.S. residents.

**What is added by this report?**

From 2020 to 2021, disparities in AADR ratios from COVID-19 decreased significantly by 14.0%–40.2% for most racial and ethnic groups, including non-Hispanic White persons, who accounted for 59.6%–65.2% of all decedents; and increased nonsignificantly (7.2%) for non-Hispanic Native Hawaiian and other Pacific Islander persons (0.2%–0.3% of all decedents) compared with non-Hispanic multiracial persons.

**What are the implications for public health practice?**

Providing effective preventive interventions, including vaccination and clinical care, to all communities in proportion to their need for these interventions is necessary to reduce racial and ethnic disparities in COVID-19 deaths.

## Footnotes

*
https://wonder.cdc.gov/mcd-icd10-provisional.html

†
https://icd.who.int/browse10/2008/en

§
https://www.census.gov/data/tables/time-series/demo/popest/2010s-national-detail.html

¶
https://www.cdc.gov/nchs/healthy_people/hp2020/health-disparities-technical-notes.htm

** 45 C.F.R. part 46, 21 C.F.R. part 56; 42 U.S.C. Sect. 241(d); 5 U.S.C. Sect. 552a; 44 U.S.C. Sect. 3501 et seq.
